# A genetically encoded toolkit for tracking live-cell histidine dynamics in space and time

**DOI:** 10.1038/srep43479

**Published:** 2017-03-02

**Authors:** Hanyang Hu, Yanfang Gu, Lei Xu, Yejun Zou, Aoxue Wang, Rongkun Tao, Xianjun Chen, Yuzheng Zhao, Yi Yang

**Affiliations:** 1 Synthetic Biology and Biotechnology Laboratory, State Key Laboratory of Bioreactor Engineering, Shanghai Collaborative Innovation Center for Biomanufacturing Technology, East China University of Science and Technology, 130 Mei Long Road, Shanghai 200237, China; 2 Optogenetics & Molecular Imaging Interdisciplinary Research Center, CAS Center for Excellence in Brain Science, East China University of Science and Technology, 130 Mei Long Road, Shanghai 200237, China; 3 Shanghai Key Laboratory of New Drug Design, School of Pharmacy, East China University of Science and Technology, 130 Mei Long Road, Shanghai 200237, China

## Abstract

High-resolution spatiotemporal imaging of histidine in single living mammalian cells faces technical challenges. Here, we developed a series of ratiometric, highly responsive, and single fluorescent protein-based histidine sensors of wide dynamic range. We used these sensors to quantify subcellular free-histidine concentrations in glucose-deprived cells and glucose-fed cells. Results showed that cytosolic free-histidine concentration was higher and more sensitive to the environment than free histidine in the mitochondria. Moreover, histidine was readily transported across the plasma membrane and mitochondrial inner membrane, which had almost similar transport rates and transport constants, and histidine transport was not influenced by cellular metabolic state. These sensors are potential tools for tracking histidine dynamics inside subcellular organelles, and they will open an avenue to explore complex histidine signaling.

As an essential amino acid and a precursor to histamine, histidine plays a vital role in human growth, metal transmission^1^, neurotransmission, and neuromodulation^2^. An abnormal level of histidine or histidine-rich proteins is also often considered as an indicator of many diseases. For example, high levels of histidine/histidine-rich proteins have been associated with phenylketonuria^3^, malaria^4^, and thrombotic disorders^5^. By contrast, low levels of histidine/histidine-rich proteins have been linked with rheumatoid arthritis^6^, epilepsy^7^, chronic kidney disease^8^, and advanced liver cirrhosis^9^. Therefore, selective and sensitive detection of histidine in living cells has become significant and indispensable, and this process facilitates the understanding of the pathogenesis for clinical therapy. Existing methods for assaying histidine, such as chromatography^10^, mass spectrometry^11^, electrochemistry^12^, and capillary electrophoresis^13^, are invasive and not suitable for studying histidine dynamics in intact individual cells. Various chemical probes have been developed to monitor histidine in living cells^14^,^15^,^16^. However, these probes are limited by subcellular location, and *in vivo* application. In addition, use concentration, incubation time and washing times of chemical probes, and even themselves also often introduce some artificial interferences.

Genetically encoded fluorescent sensors with high spatiotemporal resolution have been developed to sense various intracellular metabolites^17^. As genetically encoded proteins, these sensors can be easily introduced into living cells by DNA transfection or targeted to different subcellular compartments by tagging with organelle-specific signal peptides^18^. In addition, these sensors could also be used to establish transgenetic organisms and applied in *in vivo* research. The sensors derived from fluorescent proteins (FP) provide high sensitivity and great versatility while minimally perturbing the cells under investigation, and are leading a revolution for cell biology research^19^.

It is interesting to note that researchers have developed various sensors based on circularly permuted fluorescent proteins (cpFPs) for monitoring cellular events^20^,^21^,^22^,^23^. In these cpFPs, the original N- and C-termini are fused by a polypeptide linker, and new termini are introduced close to the fluorophore, making its fluorescence highly sensitive to the protein’s conformation. A variety of bacterial periplasmic binding proteins (PBPs) are known to sense substrates with high affinity and specificity, including amino acids, sugars, metals and inorganic ions^24^,^25^,^26^. Crystallographic studies of PBPs show that substrate binding often induces conformational changes, providing attractive scaffolds to make indicators^24^,^25^,^26^,^27^.

To overcome the disadvantages (e.g., invasiveness of sample and low spatiotemporal resolution) of existing methods, we developed a series of single fluorescent protein (FP)-based histidine sensors with various affinities and large dynamic range by combining cpYFP with a bacterial periplasmic binding protein, HisJ. These sensors, dubbed FHisJ, allow for specific, sensitive, and quantitative monitoring of histidine metabolism in various subcellular compartments of mammalian cells.

## Results

### Generation of cpYFP-based Histidine Indicators

**Figure 1:**
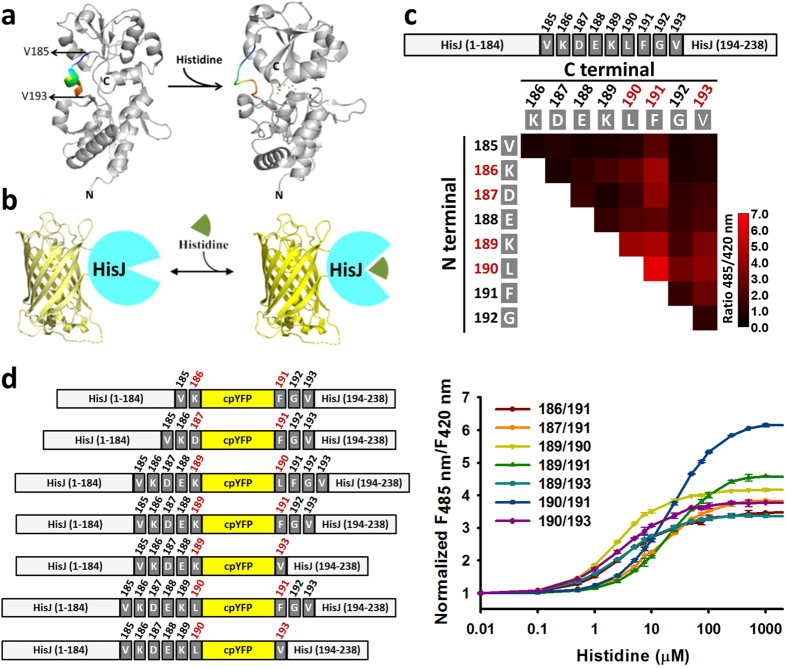
Generation of cpYFP-based histidine indicators. (**a**) Conformation of HisJ changes upon histidine binding. Crystallographic structures of histidine-free and histidine-binding HisJ based on Protein Data Bank files 2M8C and 1HSL. Histidine is indicated in ball form (green and red). The rainbow ribbon parts (residues 185–193) represent the flexible and target regions for the insertion of cpYFP to generate histidine sensors. (**b**) Design of histidine indicators. The fluorescence of cpYFP is highly sensitive to the conformational changes induced by histidine. (**c**) The 36 chimeras, in which cpYFP was inserted into the flexible linker 185–193 region of HisJ, and their fluorescence response towards 100 mM histidine. (**d**) Schematic model (left) and titration curves (right) for seven histidine indicators. Fluorescence ratios were normalized to the control condition in the absence of histidine. For (**d**), data are presented three biological replicates, and error bars represent SEM.

Various genetically encoded fluorescent sensors have been created to monitor cellular events and the microenvironment^17^,^28^. These sensors could be generally categorized into fluorescence resonance energy transfer (FRET)–based and single FP-based sensors^17^,^29^. Compared with single FP-based sensors, the dynamic ranges of FRET-based reporters usually fall into 10–150% with very few exceptions^17^,^21^, making determination of subtle differences in biochemical responses challenging^30^. We have successively developed first-generation and second-generation NADH biosensors, called Frex^31^ and SoNar^32^, respectively, by employing cpYFP. These biosensors display 800%^31^ or 1500%^32^ dynamic range, respectively, far exceeding those of other metabolite sensors^17^. To design single FP-based histidine sensors, we inserted cpYFP into a type II periplasmic binding protein HisJ, which manifests large conformational changes upon histidine binding^33^,^34^ (Fig. 1a and b). In this study, a total of 36 chimeric proteins were constructed, in which cpYFP was inserted into the flexible linker 185–193 region of HisJ (Fig. 1c). Among these proteins, seven chimeras showed 240–520% increase in the ratio of fluorescence when excited at 420 and 485 nm upon histidine addition (Fig. 1c and d and Table 1), suggesting the feasibility of the proposed engineering strategy for generating a histidine reporter. Fluorescence titration studies showed that these histidine indicators had different affinities with K_d_ of ~2.4 μM to ~22 μM (Table 1), which are suitable for measuring subcellular histidine levels in the physiological range. Considering the maximum change in fluorescence ratio and affinity constant, we chose the chimera with cpYFP inserted between Leu 190 and Phe 191 of HisJ for detailed characterization and named this chimera as Fluorescent HisJ (FHisJ) (Fig. 1d).

### *In vitro* Characterization of FHisJ

**Figure 2:**
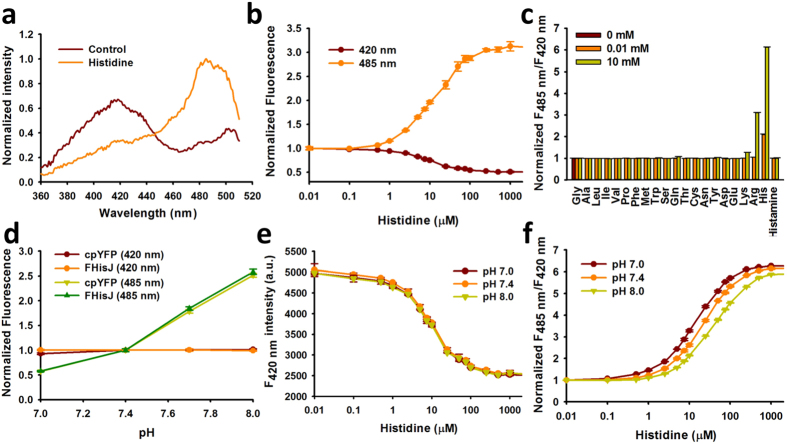
*In vitro* Properties of FHisJ Sensor. (**a**) Excitation spectra of purified FHisJ sensor in the control condition (dark red) and after addition of 1 mM histidine (orange), which was normalized to the peak intensity in the presence of 1 mM histidine. Emission was measured at 530 nm. (**b**) Fluorescence intensities of FHisJ with excitation at 420 or 485 nm in the presence of different concentrations of histidine and emission at 528 nm. Data were normalized to the initial value. (**c**) The ratio of fluorescence intensities with excitation at 485 nm divided by 420 nm (R_485/420_) in the presence of different concentrations of histidine and its analogs (other 19 amino acids and histamine). (**d**) Fluorescence intensities of FHisJ and cpYFP with excitation at 420 or 485 nm and emission at 528 nm at the indicated pH. Data normalized to the fluorescence at pH 7.4. (**e**) Fluorescence intensity of FHisJ when excited at 420 nm plotted against the histidine concentration at the indicated pH. (**f**) Histidine titration curves of FHisJ at the indicated pH. Fluorescence ratios were normalized to the control condition in the absence of histidine. For (**b**–**f**), data are presented three biological replicates, error bars represent SEM (see also Supplementary Figure 1).

The fluorescence spectrum of FHisJ is similar to those of other cpYFP-based sensors^31^,^32^ with two excitation peaks at approximately 420 and 500 nm and one emission peak near 515 nm (Fig. 2a; Supplementary Fig. 1a and b). FHisJ displayed a ~2.0-fold decrease and ~3.0-fold increase in fluorescence with excitation at 420 and 485 nm, respectively, upon histidine saturation (Fig. 2b). The opposing directional changes of this sensor, also observed in SoNar^32^, led to a 520% ratiometric change in fluorescence (Figs 1d and 2c). FHisJ possessed high selectivity toward histidine, exhibiting no apparent fluorescence changes toward 18 amino acids and histamine (Fig. 2c). FHisJ also did not respond to low concentrations of arginine (0–100 μM), although addition of 10 mM arginine resulted in a ~3.0-fold increase in the ratio of FHisJ fluorescence with excitation at 420 and 485 nm (Fig. 2c; Supplementary Fig. 1c). Quantitative measurements of intracellular arginine were rare, however, there were reports that intracellular arginine level of 90 μM^35^, and a large fraction of intracellular arginine was stored within vesicles^36^. The K_d_ of FHisJ to arginine is ~400 μM (Supplementary Fig. 1c), thus arginine should not signficantly interfere with FHisJ’s histidine sensing function in the cells. Similar to other cpYFP-based sensors^31^,^32^, FHisJ fluorescence depended on pH when excited at 485 nm (Fig. 2d), however, FHisJ fluorescence excited at 420 nm is much more pH resistant (Fig. 2d and e). In addition, FHisJ dynamic range and K_d_ were more resistant to pH changes (Fig. 2f). At modest pH fluctuations, the pH effects of FHisJ could be corrected by measuring the fluorescence of FHisJ and cpYFP in parallel, similar to Frex and SoNar^31^,^32^, because of their very similar pH responses (Supplementary Fig. 1d). Alternatively, FHisJ fluorescence could be measured with 420 nm excitation only when pH fluctuation occurred, but at the expense of reduced dynamic range of only 100% (Fig. 2e), versus 520% fluorescence ratio change when excited at 420 nm and 485 nm (Fig. 2c). For dual color ratiometric imaging, FHisJ may be fused with mCherry, a red fluorescent protein that is not sensitive to physiological pH (e.g. pH 7.0–8.0) or to histidine addition. Therefore, the ratio of FHisJ-mCherry green fluorescence excited at 420 nm and red fluorescence excited at 590 nm can be used to report histidine dynamics specifically, unaffected by intracellular pH or the sensor’s concentration. Hence, these data demonstrate that FHisJ with large dynamic range is a highy sensitive, highly selective, and intrinsically ratiometric biosensor for histidine and would be a promising tool for live-cell application.

### Subcellular Distribution of Histidine in Mammalian Cells

**Figure 3:**
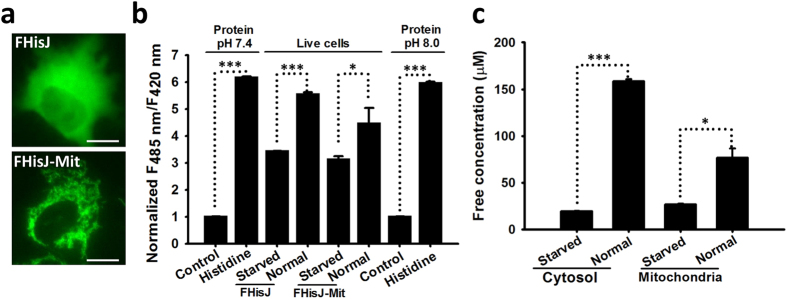
Subcellular Distribution of Histidine in Mammalian Cells. (**a**) Fluorescence images of FHisJ sensor targeted to cytosol (top) and mitochondria (bottom). Scale bar, 10 μm. (**b**) Normalized ratio of FHisJ and FHisJ-Mit fluorescence excited at 485 nm to that at 420 nm in glucose-deprived or glucose-fed cells and measured *in vitro* with a fluorescence plate reader in the presence or absence of 1 mM histidine. Cells were deprived of glucose for 2 h. Error bars represent SD. (**c**) Quantification of cytosolic and mitochondrial free-histidine level in glucose-deprived or glucose-fed cells according to the FHisJ and FHisJ-Mit fluorescence data in Fig. 3b. *p < 0.05, and ***p < 0.001. For (**b** and **c**), data are presented three biological replicates, and error bars represent SD.

We subcloned FHisJ into the pcDNA3.1/Hygro(+) mammalian expression vector and transiently expressed it in human cervical cancer HeLa cells to determine its suitability for intracellular histidine detection. Fluorescence imaging showed that FHisJ sensor with non-tagged sequences was localized exclusively in the cytosol (Fig. 3a). A duplicated cytochrome C oxidase subunit VIII signal peptide was used to lead FHisJ sensor into the mitochondrial matrix of HeLa cells to elucidate the subcellular distribution of histidine in mammalian cells (Fig. 3a). The 485 nm/420 nm fluorescence ratio of FHisJ was higher than that of FHisJ–Mit in HeLa cells when measured with a fluorescence plate reader with dual excitation (Fig. 3b). In Hela cells, the estimated resting pH was ~8.0 in the mitochondrial matrix^38^,^39^ and ~7.4 in the cytosol^38^. Fluorescence titration studies showed that the K_d_ and dynamic range of FHisJ sensor had slight differences in pH 7.4 and pH 8.0 (Fig. 2f). We carefully calibrated the effect of pH on the fluorescence of FHisJ and found that cytosolic and mitochondrial free-histidine concentrations in glucose-fed cells were ~159 and ~77 μM, respectively (Fig. 3c). However, cytosolic and mitochondrial free-histidine concentrations in glucose-deprived cells were ~20 and ~27 μM, respectively (Fig. 3c), implying that cytosolic histidine levels was more sensitive to environmental changes.

### Subcellular Transport of Histidine in Mammalian Cells

**Figure 4:**
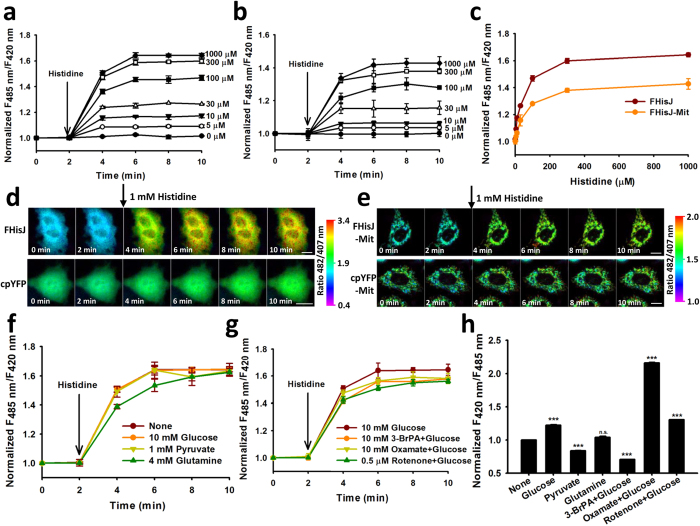
Subcellular Transport of Histidine in Mammalian Cells. (**a** and **b**) Kinetics of FHisJ (**a**) and FHisJ-Mit (**b**) fluorescence responses in Hela cells treated with exogenous histidine. (**c**) Fluorescence responses of FHisJ and FHisJ-Mit in Hela cells after histidine addition for 8 min. (**d** and **e**) Spatiotemporally resolved changes in the ratiometric fluorescence of FHisJ (**d**, top), cpYFP (**d**, bottom), FHisJ-Mit (**e**, top), and cpYFP-Mit (**e**, bottom) in sequential frames (left to right, 2 min/frame) in response to 1 mM histidine in Hela cells. Images were pseudocolored with the ratio of fluorescence excited at 482 and 407 nm. Scale bar, 10 μm. For (**a**–**e**), cells were starved in HBSS for 2 h. (**f**) Effect of different nutrients on histidine transport across plasma membrane. Cells expressing FHisJ were incubated with or without indicated nutrients for 2 h and then treated with 1 mM histidine. (**g**) Effect of glycolysis inhibitors and mitochondtial inhibitor on histidine transport across plasma membrane. Cells were treated with inhibitors for 30 min. (**h**) Effect of different nutrients, glycolysis inhibitors, and mitochondrial inhibitor on cytosolic metabolic state assessed by SoNar sensor. Experimental condition was the same as those of (**f** and **g**). ***p < 0.001. For (**a**–**c**, **f** and **g**), FHisJ or FHisJ-Mit fluorescence were corrected for pH effect by normalization with cpYFP or cpYFP-Mit fluorescence measured in parallel experiments. For (**a**–**c** and **f**–**h**), data are presented three (**a**–**c**, **f** and **g**) or six (**h**) biological replicates, error bars represent SEM. (see also Supplementary Figure 2).

Addition of exogenous histidine into the culture medium induced a rapid, dose-dependent, and saturable increase in the fluorescence ratio in the cytosol or the mitochondria of glucose-deprived HeLa cells (Fig. 4a–c). Similar results were obtained after measurement by fluorescence microscopy (Fig. 4d and e; Supplementary Fig. 2a and b), suggesting that histidine was readily transported across the plasma membrane and the inner mitochondrial membrane. By contrast, only slight changes in the fluorescence ratio were found in the cells expressing cpYFP instead of FHisJ when histidine was added to the cell culture medium (Fig. 4d and e). Thus, the possibilities of interference of fluorescence emission were excluded because of pH variations of the cpYFP domain. Furthermore, histidine transport was not affected by nutrient deprivation, nutrient feeding, glycolysis inhibition, or mitochondrial inhibition (Fig. 4f,g), which were assessed by SoNar (Fig. 4h), a highly responsive NAD^+^/NADH sensor with 1500% dynamic range^32^. This phenomenon suggested that histidine transport did not depend on cellular metabolic state. Interestingly, plasma membrane and inner mitochondrial membrane had almost similar transport rates and transport constants (K_0.5_: ~55 μM) (Fig. 4a–c).

## Discussion

In summary, we reported a series of ratiometric, and single FP-based histidine sensors with different affinities. FHisJ sensors were highly specific and highly sensitive, exhibited a large dynamic range, and could be targeted to different subcellular compartments, representing substantial improvement for live-cell histidine measurement over existing methods. It should be noted that FHisJ sensor also have its own limitations. The sensor’s affinity to histine is too high, with a dissociation constant of 22 μM, rendering the sensor largely saturated under physiological histidine concentration. Therefore the sensor’s dynamic range of in living cells is limited comparing to its *in vitro* dynamic range. Further studies are neccessary to decrease the sensor’s affinity and make it more useful to to detect changes in histidine levels in real cell biology/physiology conditions.

To our knowledge, subcellular free-histidine concentrations in living cells had not been rigorously quantified because of the lack of available non-invasive method. Our quantitative data were nearly equal to histidine levels in the plasma/serum (65–108 μM), standard tissue culture medium (97–200 μM), or whole cell (100–300 μM) as reported across several studies^40^,^41^,^42^. Michaelis constant (K_m_) of histidine uptake by mammalian cells was reported to be 100–120 μM for histidine^41^, supporting the idea that these potential histidine carriers are poised to be regulated by local histidine fluctuations.

Many transporters for histidine uptake, such as CAT1^43^, PHT1^44^, PHT2^45^, and SLC38 families^46^, have been reported, but no clear conclusion has been established. For mitochondrial histidine transport, ORC2^47^ and SLC25A29^48^ are the only known transporters that might be involved in histidine transport across the inner mitochondrial membrane. However, ORC2 transported histidine with low transport affinity (K_m_ = 1.28 ± 0.14 mM) and slow transport rate (1.2 ± 0.2 mmol/min/g protein)^47^. Thus, ORC2 might not serve as a mitochondrial histidine carrier in HeLa cells. SLC25A29 could also transport histidine into the mitochondria, but it was mainly responsible for mitochondrial arginine and lysine uptake^48^, with transport affinities (K_0.5_) of 0.42 ± 0.04 and 0.71 ± 0.10 mM, respectively. The net import of histidine into the cytosol or mitochondria is necessary for the synthesis of histamine or intramitochondrially translated proteins^2^,^49^. Therefore, further study is of vital importance to address these issues on subcellular histidine carrier.

The superior properties of FHisJ sensors facilitated the use of these sensors for real-time tracking of cellular histidine fluctuations in individual mammalian cells using fluorescence imaging. FHisJ sensors are also compatible with high-throughput screening of candidate genes or drugs affecting uptake, efflux, and metabolism of histidine using microplate readers and flow cytometers. We believe that FHisJ sensors should faciliate a more complete understanding of the pathophysiological relevance of organellar histidine metabolism because of the significance of histidine in physiological and pathological conditions. These sensors are good alternatives to existing methods for intracellular histidine detection.

## Materials and Methods

### Plasmid construction

The gene encoding HisJ (positions 67–780 relative to ATG) was amplified from *Escherichia coli* genomic DNA by PCR with the primers P1 (CCCGGATCCGATGGCGATTCCGCAAAAC) and P2 (CCCAAGCTTTTAGCCACCATAAACAT) and cloned into the pRSET-B vector (Invitrogen). The cDNA of cpYFP^31^,^32^ was amplified by PCR with the primers P3 (TACAACAGCGACAACGTC) and P4 (GTTGTACTCCAGCTTGTG). The cDNA of FHisJ consisting of HisJ fused to cpYFP was generated by an overlapping PCR and cloned into the BamHI/HindIII sites of pRSET-B vector for bacterial expression. The entire coding sequences of FHisJ were subcloned into pcDNA3.1 Hygro(+) (Invitrogen) behind a Kozak sequence for mammalian expression. For mitochondrial matrix targeting, the duplicated mitochondrial targeting signal^50^,^51^,^52^,^53^ MSVLTPLLLRGLTGSARRLPVPRAKIHSLGDLSVLTPLLLRGLTGSARRLPVPRAKIHSLGD was inserted at the N-terminus of FHisJ.

### Protein Expression and Purification

*E. coli* JM109 (DE3) cells carrying the pRSETb-FHisJ expression plasmid were grown in Luria-Bertani (LB) media containing 100 μg/ml ampicillin at 37 °C until the cultures reached approximately 0.4–0.6 OD. The expression of His_6_-tagged proteins was induced by the addition of 0.1 mM IPTG, and the cells were cultured at 18 °C overnight. Bacteria were then centrifuged at 4000 × *g* for 30 min at 4 °C. The cell pellets were suspended in buffer A (20 mM sodium phosphate, 500 mM sodium chloride, and 20 mM imidazole, pH 7.4) and lysed via ultrasonication. Proteins were purified using a Ni–NTA His SpinTrap column. After washing with 2 column volumes of wash buffer (buffer A containing 50 mM imidazole), the proteins were eluted from the resin using buffer B (20 mM sodium phosphate, 500 mM sodium chloride, and 500 mM imidazole, pH 7.4). The protein preparations were then desalted and exchanged into 20 mM sodium phosphate buffer (pH 7.4) before assay.

### *In vitro* Characterization of FHisJ

We stored the purified protein at −20 °C until use. Excitation and emission spectra of recombinant fluorescent sensor proteins was measured as previously described^31^,^32^. The purified sensor protein was placed into a cuvette containing 20 mM sodium phosphate buffer (pH 7.4). Fluorescence was measured using a Cary Eclipse fluorescence spectrophotometer (Agilent). Excitation spectra was recorded with an excitation range from 350 nm to 510 nm and emission wavelength of 530 nm. The emission range for the emission spectra was 505–550 nm, while the excitation wavelength was 490 nm. Readings were taken every 1 nm for excitation spectra and every 3 nm for emission spectra.

For substrate titration, the protein was diluted in 20 mM sodium phosphate buffer (pH 7.4) to a final concentration of 1 μM. The fluorescence value of FHisJ sensor, in the absence of a substrate, was measured by a filter-based Synergy 2 Multi-Mode microplate reader using 420 BP 10 nm or 485 BP 20 nm excitation and 528 BP 20 nm emission band-pass filters (BioTek). The stock solution of histidine was also prepared in HEPES buffer (pH 7.4). Each assay was performed with 10 μl amino acids and 90 μl proteins in black 96-well flat bottom plate. Fluorescence intensity was read immediately after addition of substrate.

### Cell Culture and Transfection

HeLa cells were grown in high-glucose DMEM (HyClone) with 10% FBS (HyClone) and cultured at 37 °C in a humidified atmosphere of 95% air and 5% CO_2_. For DNA transfection, we typically used 0.1 μg of plasmids with 0.3 μl of Lipofectamine 2000 for each well of a 96-well plate according to the manufacturer’s protocol.

### Fluorescence Microscopy Imaging

Fluorescence microscopy imaging was performed as previously described^32^. Briefly, cells were plated on a 35 mm glass bottom dish (segmented) and observed after 30–36 h transfection. Glucose-free HBSS (10 mM HEPES, 136.7 mM NaCl, 5.4 mM KCl, 0.35 mM Na_2_HPO_4_, 0.44 mM KH_2_PO_4_, 4.2 mM NaHCO_3_, 1.26 mM CaCl_2_, 0.81 mM MgSO_4_, pH 7.4) was used to replace the growth medium before imaging. Images were acquired using a high-performance fluorescent microscopy system equipped with Nikon Eclipse Ti-E automatic microscope, monochrome cooled digital camera head DS-Qi1 Mc-U2 and the highly stable Sutter Lambda XL light source. A Plan Apo 20 × 0.75 NA objective was utilized. Cells were maintained at 37 °C under a humidified atmosphere using a CO_2_ incubator (Tokai Hit). Dual-excitation ratio imaging was performed using 407 BP 17 nm or 482 BP 35 nm band-pass excitation filters (Semrock) and a 535 BP 40 nm emission filter altered by a Lambda 10-XL filter wheel (Shutter Instruments). Images were captured using 1280 × 1024 format, 12 bit depth, and 2 × gain. Raw data were exported to ImageJ software as 12 bit TIF for analysis. The pixel-by-pixel ratio of the 482 nm excitation image by the 407 nm excitation image of the same cell was used to pseudocolor the images in the HSB color space. The RGB value (255, 0, 255) represents the lowest ratio, and red (255, 0, 0) represents the highest ratio. Color brightness is proportional to the fluorescent signals in both channels.

### Live-cell Fluorescence Measurement Using Microplate Reader

HeLa cells were seeded in a black 96-well flat-bottom plate and transfected with the plasmid DNA of FHisJ and cpYFP for 30–36 h. Cells were rinsed twice, incubated in HBSS containing 10 mM HEPES (pH 7.4), and then maintained at 37 °C during the measurement. Dual-excitation ratios were obtained by a Synergy 2 Multi-Mode Microplate Reader (BioTek) with excitation filters 420 BP 10 nm and 485 BP 20 nm and emission filter 528 BP 20 nm emission for both excitation wavelengths. Fluorescence values were background corrected by subtracting the intensity of HeLa cell samples not expressing sensors. Unless otherwise indicated, glucose was not maintained in the buffer.

### Calibration of Intracellular Free Histidine Levels

Intracellular free histidine levels were measured after calibration of FHisJ fluorescence in live cells with that of recombinant FHisJ protein as described previously^31^. Ratiometric measurement of FHisJ fluorescence is possible for FHisJ expressed in the cytosol or mitochondria using the microplate reader. In these experiments, samples containing equal numbers of FHisJ-expressing cells or untransfected (control) cells were measured. The background values (from control cells) were subtracted from those of FHisJ-expressing cells. This correction basically eliminated the interference of not only the autofluorescence of the microplate but also the autofluorescence of the cells. The equation is as follows:


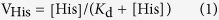



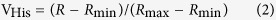



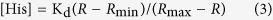


V_His_ is the fraction of FHisJ sensor bound to histidine, and [His] is the free concentration of histidine. *K*_d_ represents K_His_, which is the ratio of histidine at which the response of the sensor is half-maximal. *R*_min_ and *R*_max_ represent the F_485 nm_/F_420 nm_ ratio of the recombinant FHisJ protein in the absence or presence of 1 mM Histidine, respectively. *R* represents the F_485 nm_/F_420 nm_ ratio of the cells.

### Statistical Analysis

Data are presented either as a representative example of a single experiment repeated at least in triplicate or as three or more experiments. Data obtained are represented as mean values ± SD or mean values ± SEM. All p values were obtained using unpaired two-tailed Student’s t test. Values of p < 0.05 were considered statistically significant (*p < 0.05, **p < 0.01, and ***p < 0.001).

## Additional Information

**How to cite this article:** Hu, H. *et al*. A genetically encoded toolkit for tracking live-cell histidine dynamics in space and time. *Sci. Rep.* **7**, 43479; doi: 10.1038/srep43479 (2017).

**Publisher's note:** Springer Nature remains neutral with regard to jurisdictional claims in published maps and institutional affiliations.

## Supplementary Material

Supplementary Figures

## Figures and Tables

**Table 1:** Properties of histidine sensors.

Insertion site	Sensor name	Detection mode (fluorescent protein)	*K*_*d*_ (~μM)	Dynamic range (%)	Detection range (μM)
186/191	FHisJ_4.0μ_	Ratiometric (cpYFP)	4.0	260	0.16–100
187/191	FHisJ_13μ_	Ratiometric (cpYFP)	13	280	0.5–350
189/190	FHisJ_2.4μ_	Ratiometric (cpYFP)	2.4	320	0.1–70
189/191	FHisJ_21μ_	Ratiometric (cpYFP)	21	360	0.6–730
189/193	FHisJ_3.4μ_	Ratiometric (cpYFP)	3.4	240	0.15–80
**190/191**	**FHisJ**	**Ratiometric (cpYFP)**	**22**	**520**	**0.4–1100**
190/193	FHisJ_3.2μ_	Ratiometric (cpYFP)	3.2	280	0.1–80
