# Supplementary material for: A genetically encoded toolkit for tracking live-cell histidine dynamics in space and time
Source: Sci Rep. 2017 Mar 2;7:43479. doi: 10.1038/srep43479 (PMC5333150; doi:10.1038/srep43479)
Supplement: Supplementary Figures [file srep43479-s1.pdf]

Supporting Information

A genetically encoded toolkit for tracking live-cell histidine dynamics in space and time

Hanyang Hu[1,2,3], Yanfang Gu[1,2,3], Lei Xu[1,2], Yejun Zou[1,2,3], Aoxue Wang[1,2,3], Rongkun Tao[1,2,3], Xianjun Chen[1,2,3], Yuzheng Zhao[1,2,3,]*, Yi Yang[1,2,]*

[1]*Synthetic Biology and Biotechnology Laboratory, State Key Laboratory of Bioreactor Engineering, Shanghai Collaborative Innovation Center for Biomanufacturing Technology, East China University of Science and Technology, 130 Mei Long Road, Shanghai 200237, China.*

[2]*Optogenetics & Molecular Imaging Interdisciplinary Research Center, CAS Center for Excellence in Brain Science, East China University of Science and Technology, 130 Mei Long Road, Shanghai 200237, China.*

[3] *Shanghai Key Laboratory of New Drug Design, School of Pharmacy, East China University of Science and Technology, 130 Mei Long Road, Shanghai 200237, China.*

**Correspondence should be addressed to Y.Zhao (yuzhengzhao@ecust.edu.cn) and Y.Y. (yiyang@ecust.edu.cn)*

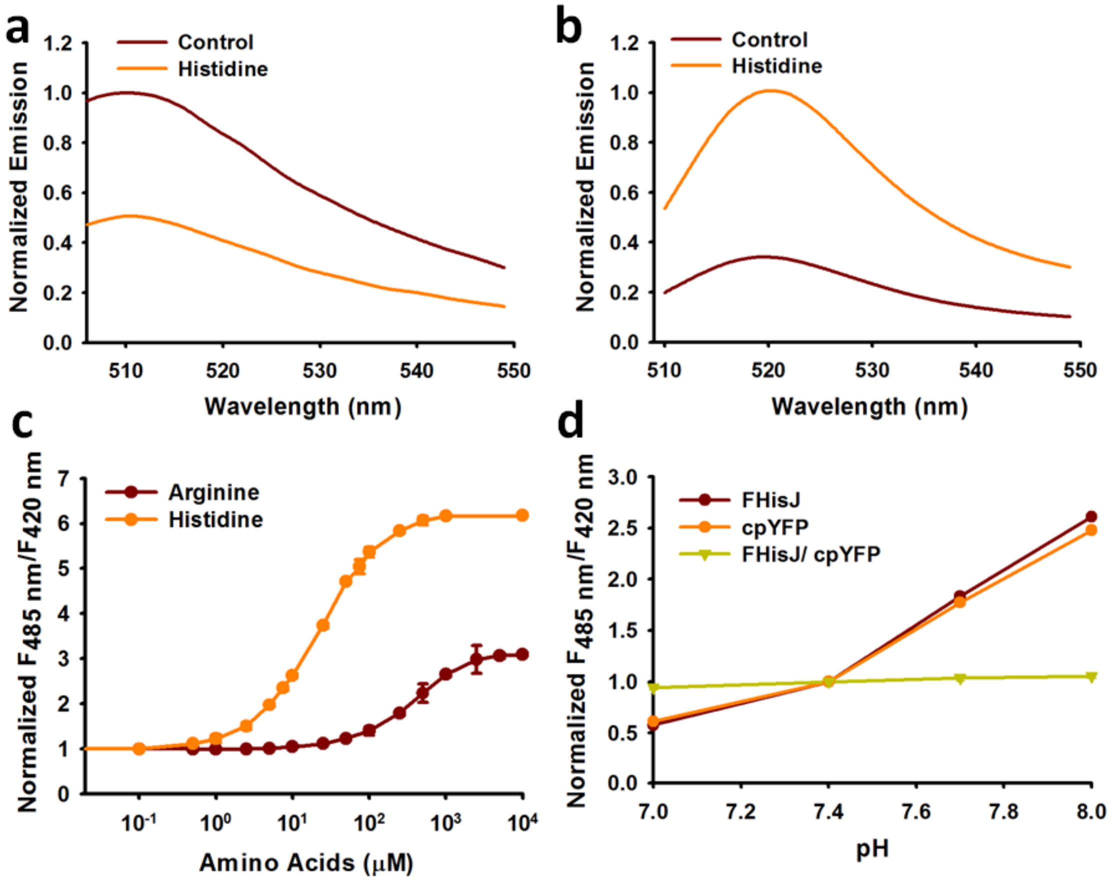

Supplementary Figure 1. (a and **b)** Emission spectra of purified FHisJ in the control condition (dark red) and after addition of 1 mM histidine (orange), which was normalized to the peak intensity. Excitation was fixed at 420 (a) and 490 nm (b), respectively. **(c)** Responses of FHisJ to different concentrations of histidine and arginine. **(d)** pH-dependency of the excitation ratio 485/420 nm of FHisJ and cpYFP. Data normalized to the fluorescence ratio at pH 7.4. Error bars represent SEM.

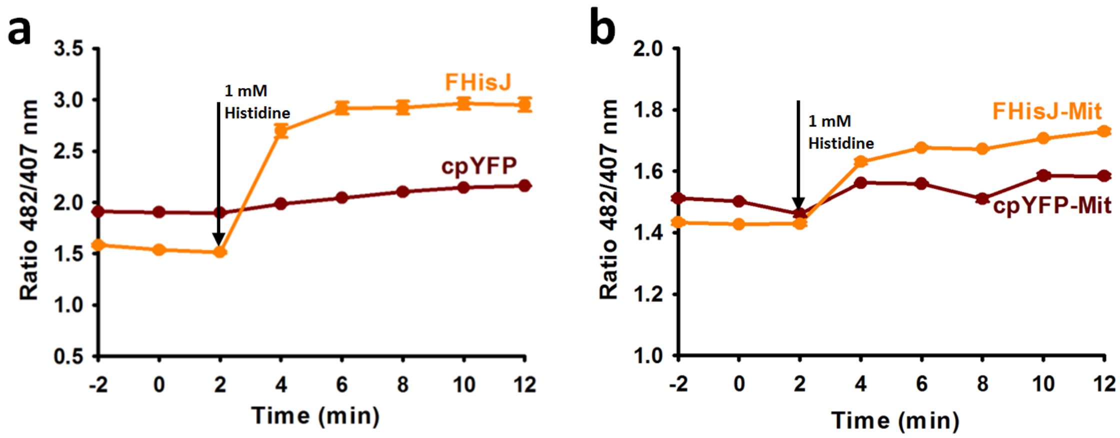

Supplementary Figure 2. (a and **b)** Kinetic course of averaged FHisJ, cpYFP (a), FHisJ-Mit or FHisJ-cpYFP (b) ratio changes in response to 1 mM histidine in Hela cells measured by fluorescent microscopy. Data were from Fig. 4d and 4e, respectively. Error bars represent SD.
